# Metabolizable Energy Value of Chickpeas and Lentils in the Human Diet: A Randomized Controlled Trial

**DOI:** 10.3390/nu17172725

**Published:** 2025-08-22

**Authors:** Janet A. Novotny, Theresa Henderson, David J. Baer

**Affiliations:** Beltsville Human Nutrition Research Center, Agricultural Research Service, United States Department of Agriculture, Beltsville, MD 20705, USA; theresa.henderson@usda.gov (T.H.); david.baer@usda.gov (D.J.B.)

**Keywords:** chickpeas, lentils, metabolizable energy, calories

## Abstract

Background/Objectives: Accurate knowledge of the energy (calorie) value of foods is important for food labeling, food policy, and diet planning to support health. Virtually no data are available on the energy values of chickpeas and lentils,—two pulses that help control blood glucose and body weight. The objective of this study was to measure the metabolizable energy value of chickpeas and lentils when fed as part of a diet and compare those values to Atwater values. Methods: A randomized controlled crossover intervention was conducted to measure the energy value of chickpeas and lentils in humans (*n* = 18). Total 7-day fecal and urine collections were conducted after a 10-day adaptation to the controlled diet. Results: The metabolizable energy (ME) of the chickpeas is 515 ± 17 kJ/serving (123 ± 4 kcal/serving) (serving mass = 85.5 g), which is 10.4% lower (*p* = 0.002) and 8.0% lower (*p* = 0.02) than the ME calculated using Atwater General or Specific Factors, respectively. The metabolizable energy of the lentils is 498 ± 17 kJ/serving (119 ± 4 kcal/serving) (serving mass = 98.5 g), which is 16.0% lower (*p* < 0.0001) and 13.6% lower (*p* = 0.003) than the ME calculated using Atwater General or Specific Factors, respectively. Conclusions: Using Atwater Factors to calculate the metabolizable energy value of chickpeas and lentils overestimates their available calories.

## 1. Introduction

Providing consumers with accurate information on the energy content of their food is important for those interested in maintaining or achieving a healthy body weight. Moreover, this information is required for food labeling, and accurate energy content of foods is important for food policies.

The approach for estimating usable energy in a food item involves determining the protein, fat, and carbohydrate content of the food, then multiplying the mass of each macronutrient by the energy density of that macronutrient. The original energy density values (Atwater General Factors) were estimated based on a mixed diet then later refined to reflect specific food groups (Atwater Specific Factors) [1]. However, because the digestibility and bioavailability of macronutrients can differ by food source, the Atwater Factors do not always provide an accurate representation of available energy (termed metabolizable energy). Previous studies in our lab have demonstrated that the Atwater General and Specific Factors overestimate metabolizable energy from tree nuts. Our previous studies were conducted with almonds, cashews, pistachios, and walnuts, and the mean Atwater overestimation for individual tree nuts ranged from 5 to 32% [2,3,4,5,6,7,8,9]. This overestimation is likely due to the limited digestibility resulting from the high content of fiber and cell wall material. Similarly to tree nuts, legumes are also high in fiber, and macronutrients are encapsulated in cell walls that may inhibit digestibility. Nutritionally, legumes provide protein, fiber, and other nutrients such as folate. Chickpea and lentil consumption specifically has increased significantly in the past 10–15 years in the USA [10,11]. The nutritional importance of lentils and chickpeas along with their increasing popularity provide rationale to investigate the accurate energy value of lentils and chickpeas when consumed in the human diet.

The objective of this project was to measure, in humans, the energy value of chickpeas and lentils and to compare their values to those calculated using Atwater Factors. Based on the inaccuracies of the Atwater system for foods high in fiber and cell wall material, we hypothesized that the measured energy value of chickpeas and lentils would be less than the calculated value.

## 2. Materials and Methods

### 2.1. Study Design and Controlled Feeding

A randomized controlled trial was conducted with 18 healthy adults (12 females, 6 males) in a crossover design to determine the metabolizable energy content of chickpeas and lentils. The trial included three dietary treatments: (1) a base diet without chickpeas or lentils, (2) the base diet with chickpeas, and (3) the base diet with lentils. Volunteers were randomly assigned to one of 6 treatment sequences (one male and two females were assigned to each treatment sequence), and each volunteer consumed all three dietary treatments.

Except for the chickpeas and lentils, the foods in all three diets were identical. For the diets containing lentils or chickpeas, the amount of every base diet food item was reduced by a proportional amount such that the estimated energy intake of all diets was similar when the chickpeas and lentils were included, in an attempt to keep the diets as isocaloric as possible. Several varieties of chickpeas or lentils were provided in the chickpea- and lentil-containing diets. The varieties and their proportion were based on lentil and chickpea production in the United States (Table 1). Dry chickpeas and lentils were prepared prior to the start of feeding using standardized methods for cooking. In preparation for feeding, cooked lentils were mixed (Table 1) and frozen, and cooked chickpeas were mixed with the canned chickpeas (Table 1) and frozen. The amount of chickpeas or lentils included in the two legume-containing diets was 1 cup of cooked chickpeas (171 g) or 1 cup of cooked lentils (197 g) per day per 8.37 MJ (2000 kcal). The amount of chickpeas or lentils incorporated into the diet was proportional based on energy intake required to maintain body weight. For example, a volunteer requiring 16.7 MJ/day (4000 kcal/day) to maintain body weight would receive 342 g of cooked chickpeas and 394 g of cooked lentils, which is twice as much as a volunteer requiring 8.37 MJ/day (2000 kcal/day). To assess weight maintenance, body weight was measured Monday through Friday before breakfast.

Monday through Friday, breakfast and dinner were consumed at the Center under supervision of research staff. Lunches and meals for weekends were prepared at the Facility and packed for consumption off-site. Chickpeas or lentils were consumed at breakfast and dinner, under staff supervision.

The research protocol and informed consent were reviewed and approved by Advarra (protocol #Pro00031506, Columbia, MD, USA, approval date: 2 January 2019) and the trial was registered at Clinicaltirals.gov (NCT03779971, registration date: 14 December 2018). All participants provided written informed consent prior to screening.

### 2.2. Participants

Volunteers were recruited by email and direct mail from the area surrounding the Beltsville Human Nutrition Research Center (Beltsville, MD, USA). Potential volunteers were required to attend a meeting where details of the study were provided, and informed consent obtained. To determine eligibility, volunteers completed a screening which included a blood cardiometabolic profile, complete blood count, urine analyses, height, weight, and a self-reported medical history. In order to participate in the study, volunteers could not be younger than 25 years nor older than 75 years; have a body mass index below 19 or above 38 kg/m^2^; have known (self-reported) allergy or adverse reaction to study foods; have given birth during the previous 12 months or who are pregnant/lactating or who plan to become pregnant during the study; have a history of bariatric surgery or nutrient malabsorption disease (such as celiac disease), Crohn’s disease, diabetes, or metabolic disorders that may interfere with the study; have a history of certain cancer diagnoses or treatment in the last 3 years; smoke, vape, or use of tobacco products in the past 6 months; have suspected or known strictures, fistulas or physiological/mechanical GI obstruction; use of certain medications or supplements (prescription or over-the-counter) that may interfere with the study objectives, including blood thinning medications; be unable or unwilling to give informed consent or communicate with study staff; self-report of alcohol or substance abuse within the past 12 months and/or current treatment for these problems (long-term participation in Alcoholics Anonymous is not an exclusion); or have other medical, psychiatric, or behavioral factors that in the judgment of the Principal Investigator may interfere with study participation or the ability to follow the intervention protocol.

### 2.3. Biospecimen and Diet Collection and Analysis

Each diet was consumed for a total of 17 days. After 10 days of adaptation to the controlled diet, a capsule containing approximately 30 mg of Brilliant Blue food dye was consumed on the morning of the 10th day. Volunteers were instructed to collect all voided urine and feces. Urine was collected in 4 L jugs (pre-weighed and containing 10 g boric acid) and stored with ice packs until returned to the Center. Fecal samples were collected in plastic storage bags and stored in Styrofoam coolers with dry ice until they were returned to the Center. Volunteers were instructed to record the date and time of each bowel movement on the storage bags. At the end of 7 days, a second 30 mg capsule of Brilliant Blue was consumed. Volunteers continued to collect feces until a staff member confirmed the appearance of the food dye marker in the feces and informed the volunteer that they could cease fecal collections.

Diets were collected during each treatment period and prepared as though they were to be consumed (i.e., bread toasted). The diets were processed in a Waring blender into a slurry, then freeze-dried. The dried material was processed in a food processor to produce a homogeneous powder. A proportion of each day’s diet (15%) was pooled and mixed to create a composite of the week’s food for analyses. A total of 4 samples of each diet (base, base + chickpea, base + lentil) were collected during the study. The chickpeas and lentils were collected over the week and processed separate from their base diets.

Fecal samples were pooled based on the presence of the markers in the feces. The fecal samples were weighed (wet weight), freeze dried and then reweighed to determine dry weight. After drying, they were pulverized to create a homogeneous powder which was frozen until subsequent analyses.

When returned to the Center, jugs containing urine were weighed and the daily weight of urine was determined as the difference between full weight and tare weight. A subsample of urine was collected into 5 mL cryovials, and frozen for subsequent pooling and analyses.

Urine, fecal, and diet samples were analyzed in duplicate for gross energy by bomb calorimetry (Parr 6400 Automatic Isoperibol Calorimeter, Moline, IL, USA), for nitrogen by combustion (Leco TruMac Nitrogen Determinator, St. Joseph, MI, USA), for ash by combustion (muffle furnace), for crude fat by petroleum ether extraction (Ankom XT15 Extractor, Macedon, NY, USA), and total dietary fiber (Ankom, TDF Fiber Analyzer, Macedon, NY, USA, AOAC method 991.43). Dietary protein was determined as nitrogen x 6.25. Total carbohydrate was determined by difference.

The metabolizable energy of chickpeas and lentils was determined using the previously published method [3,4,5,6,7], as follows:

(1) $$ME\;Pulse\;(\frac{kJ}{g})=\frac{\left[MEI\;Pulse\;diet\right]-\left[\left(GEI\;Pulse\;diet-GEI\;Pulse\right)\times \left(\frac{MEI\;Base\;diet}{GEI\;Base\;diet}\right)\right]}{\Delta \mathrm{Pulse}\;\mathrm{Intake}\;}.$$

where ME is the metabolizable energy of the pulse (chickpea or lentil), MEI is metabolizable energy intake, and GEI is gross energy intake.

Apparent digestibility (percent) of dry matter, energy, fat, and carbohydrate were calculated as

(2) $$Nutrient\;or\;energy\;digestibility\;(\%)\;=\;\left[\frac{Intake-Excreted}{Intake}\right]\times 100.$$

Converting nitrogen to protein using a factor, such as 6.25, is likely not valid for microbial proteins collected from feces. Thus, nitrogen balance instead of protein digestibility is best suited for measuring nitrogen disappearance through the gastrointestinal tract. Nitrogen balance (g/day) was calculated as

(3) $$Balance\;nitrogen\;\left(\frac{g}{d}\right)\;=\;Intake\;nitrogen-Fecal\;nitrogen-Urine\;nitrogen.$$

### 2.4. Sample Size, Randomization, and Statistical Analysis

The sample size was determined based on an expected of 10% between the measured and calculated energy value of chickpeas and lentils, a standard deviation of the difference of 0.6 kcal/g measured in similar studies [3,4,5,6,7], a power of 80% for a 1-sided test with an alpha = 0.05. This calculation provided a sample size of *n* = 16. The sample size recruited was increased by *n* = 2 to account for possible dropouts (based on previous dropout rates) for a total sample size of *n* = 18. Subjects were randomly assigned to one of six treatment sequences blocked by sex (one male randomly assigned to each unique sequence and two females randomly assigned to each unique sequence). Because of the nature of the dietary intervention, volunteers were not blinded to their treatment. Staff collecting and processing samples, and analysts and statisticians were blinded to treatments. Statistical analyses were performed using R (version 4.0.4) and R Studio (version 1.4.1106). Mixed models analysis of variance (using lme4 package and lme function) was performed using subject (volunteer) as a random variable and the following as fixed effects treatment (control, chickpea or lentil), treatment sequence (1..6), treatment period (1..3), age, sex (M, F), BMI, and interactions of treatment x period, treatment x sequence, and treatment x sex. For each variable with a significant treatment effect, lsmeans were compared using Tukey contrasts. To determine if the measured energy value of chickpeas and lentils is different from the calculated energy value (using Atwater General Factors and Atwater Specific Factors), a two-tailed Student’s paired *t*-test was calculated.

## 3. Results

**Figure 1:**
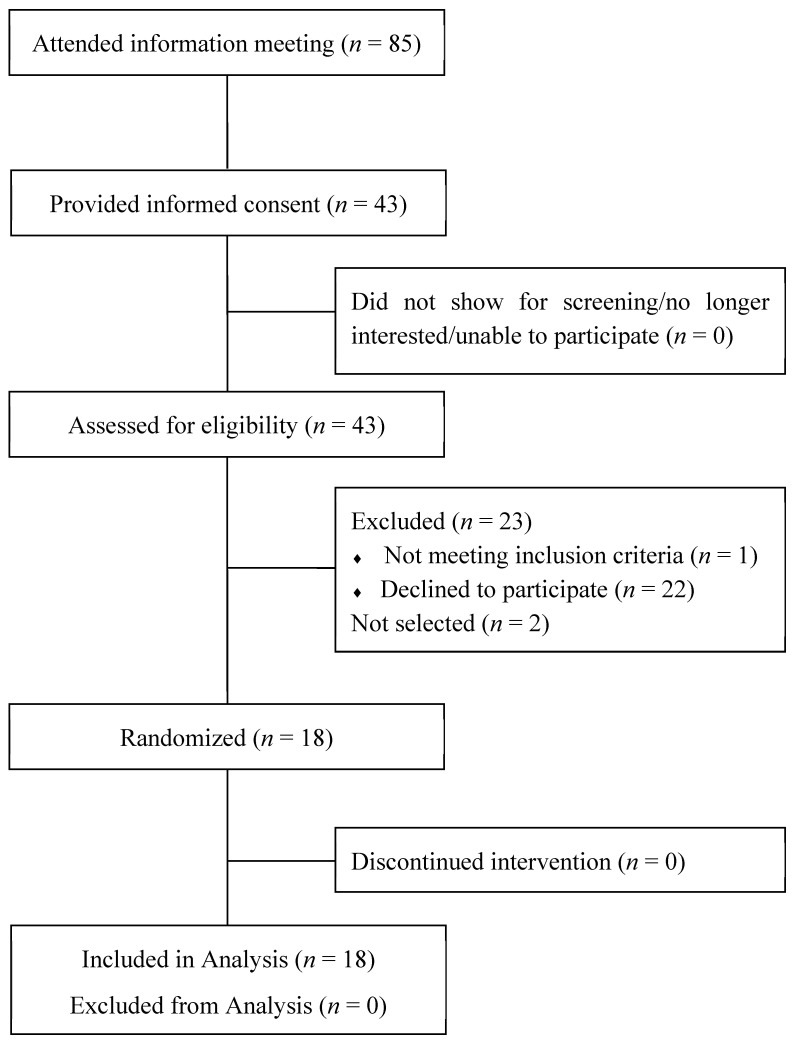
CONSORT flow diagram describing participant recruitment, selection, randomization, and completion.

Figure 1 shows the participation in the intervention study to determine the available energy in chickpeas and lentils. In total, 1 volunteer did not meet the eligibility criteria, 22 volunteers declined to participate, and 2 volunteers were not selected. Eighteen volunteers were randomized to a treatment sequence, and all randomized subjects completed the intervention. For the randomized volunteers (12 females, 6 males), mean (±SEM) age was 55.8 ± 3.4 y, mean body mass was 82.8 ± 5.0 kg and 90.6 ± 5.1 kg, for females and males, respectively, and mean body mass index (BMI) was 30.9 ± 1.7 kg/m^2^ and 28.6 ± 1.4 kg/m^2^ for females and males, respectively. At the end of each treatment period, there were no differences (*p* > 0.9) in body weight (mean body weight for females 80.8 ± 2.6 kg, and for males 96.6 ± 3.7 kg). These end-of-treatment body weights confirm that the volunteers were in weight maintenance.

For variables related to food intake and fecal output, there were interactions between sex and treatment (due to males consuming more than females). These data are presented for each sex. There were no sequence, period x treatment or sequence x treatment interactions signifying that there were no carryover effects in this crossover design study.

The amount of food consumed (dry matter intake) and energy and nutrient intake were different among the three diets (Table 2). In general, food intake, energy intake and macronutrient intake were lowest when volunteers consumed the control diet and highest when they consumed the lentil diet. These effects were similar for males and females. While these effects were highly significant (overall *p* < 0.0001), the differences in intake were small and not clinically meaningful. For example, the difference in energy intake between the control diet and lentil diet (two extremes) was 2% (for both males and females). The difference in total food intake (dry matter) was also significant, but again, less than a 5% difference between the two diet extremes (base diet and lentil diet). The differences are a reflection on the precision of our diet preparation and methodology, leading to very low variance. On the other hand, there were larger differences in protein, fat, and total carbohydrate intake among the three diets. These differences reflect the compositional differences in the chickpeas and lentils relative to the base diet and are expected. Compared to the base diet, protein, total carbohydrate and total dietary fiber intakes were higher and fat intake lower when volunteers consumed the diets containing chickpea and lentils.

There were no differences in the mean number of daily bowel movements among the three treatments (the mean number of daily bowel movements ranged from 1.0 to 1.3, Table 3). However, the amount of daily fecal excretion (dry matter) did increase (*p* < 0.0001) when chickpeas and lentils were included in the diet compared to control (Table 3). These results were consistent for both males and females.

Fat and total dietary fiber excretion were not different among treatments, while energy, nitrogen and carbohydrate excretion were higher after consumption of the pulse containing treatments compared to the base diet. The pattern of energy and nutrient excretion was similar for males and females. Because of the changes in intake and excretion, apparent total dry matter, gross energy, and total carbohydrate digestibilities were lower on the diets containing chickpeas and lentils compared to the base diet (Table 4). There was no difference among the three diets with respect to fat and total dietary fiber digestibility and nitrogen balance.

The measured metabolizable energy values for both pulses were lower than the calculated values using the Atwater Factors (Table 5). The mean (± SEM) metabolizable energy of the chickpeas was 515 ± 17 kJ/serving (123 ± 4 kcal/serving) (serving mass = 85.5 g). Using the composition of the chickpeas in this study, the metabolizable energy value is 10.4% lower (*p* = 0.002) than the metabolizable energy calculated using Atwater General Factors, and 8.0% lower (*p* = 0.02) than the metabolizable energy calculated using Atwater Specific Factors (and determining protein as N x 6.25). The metabolizable energy of the lentils was 498 ± 17 kJ/serving (119 ± 4 kcal/serving) (serving mass = 98.5 g). Using the composition of the lentils in this study, the metabolizable energy value is 16.0% lower (*p* < 0.0001) than the metabolizable energy calculated using Atwater General Factors, and 13.6% lower (*p* = 0.003) than the metabolizable energy calculated using Atwater Specific Factors (and determining protein as N x 6.25).

**Figure 2:**
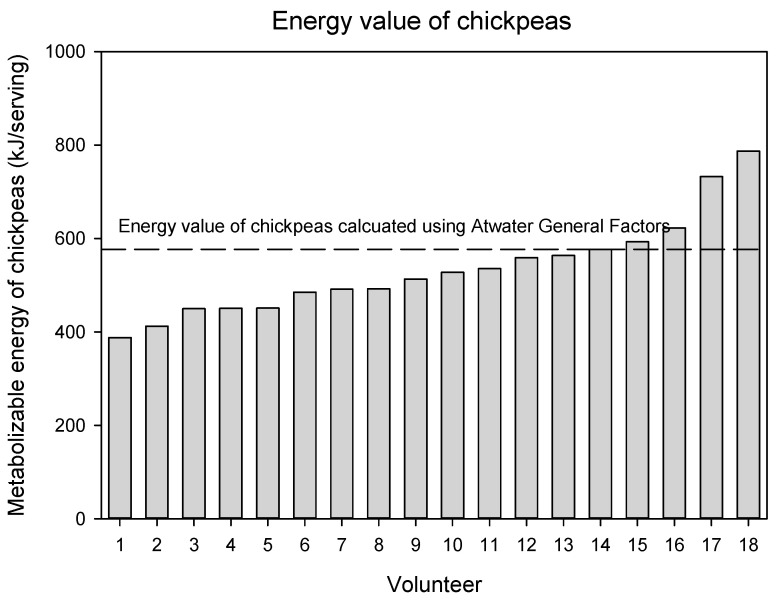
Metabolizable energy value of chickpeas for each individual volunteer. The dashed line represents the metabolizable energy value calculated using Atwater General Factors.

**Figure 3:**
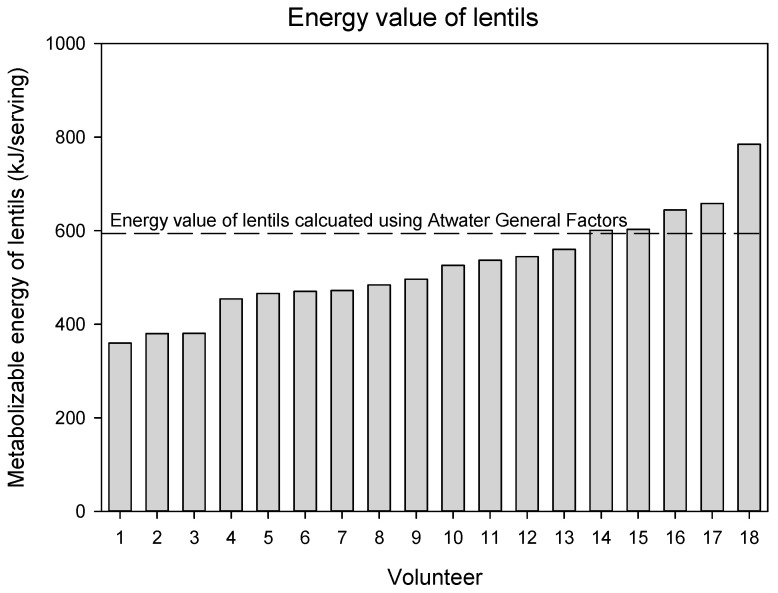
Metabolizable energy value of lentils for each individual volunteer. The dashed line represents the metabolizable energy value calculated using Atwater General Factors.

The metabolizable energy value of chickpeas and lentils for each volunteer are presented in Figure 2 and Figure 3. Notably, there were three volunteers whose measured energy value for chickpeas was slightly higher than the calculated energy value. With respect to lentils, there were two volunteers whose measured energy value was similar to the calculated energy value but for all others, the energy value was lower.

## 4. Discussion

The present study is the first to directly measure the dietary energy value of chickpeas and lentils. There is no evidence that chickpeas were fed in any of the studies conducted in the historical work of Atwater or his colleagues. There are a few studies that have reported on lentils but those focused on protein apparent digestibility and not energy [12]. In this study, the metabolizable energy value of chickpeas and lentils was measured and found to be significantly lower than those values calculated with Atwater General Factors or Atwater Specific Factors. The magnitude of the difference was greater for chickpeas than lentils. The Atwater Specific Factors compared to the Atwater General Factors were closer to measurement values (but still significantly higher than measured).

These results of overestimation of metabolizable energy by the Atwater calculation method are in accord with our previous findings for tree nuts. In those studies, we found that the Atwater method overestimated metabolizable energy by 32% for almonds [6], by 19% for cashews [3], by 5% for pistachios [7], and by 27% for walnuts [5].

Given the global concerns about the incidence of obesity, leading health organizations such as the WHO have suggested that consumption of legumes can help control or reduce obesity [13]. Some studies have demonstrated an inverse relationship between pulse consumption and body weight. No studies focus on lentils and body weight, but three studies include chickpeas in these interventions (most are studies of beans) [14,15,16]. In studies of chickpeas, body weight did not change over a 5- or 6-week period for the chickpea intervention arms. Notably, body weight for these studies was not the primary outcome, and in at least one study, there was guidance given to subjects to maintain their body weight [15]. While individual data on chickpeas and lentils is limited, systematic reviews demonstrate an inverse relationship between pulse consumption and body weight [17,18,19]. Various mechanisms have been proposed to explain the observed inverse relationship, and include the satiating properties of pulses, changes in gastric emptying, improved blood glucose concentrations, specific nutrients such as fiber and protein affecting intake, the presence of protease and amylase inhibitors as well as phenolic compounds [18,19]. These results add supporting evidence for chickpeas and lentils as part of a health-promoting diet with lower metabolizable energy values than previously understood.

The cell wall structure of plants is in part responsible for limiting nutrient and energy availability [20,21]. In a previous study, milling was used to disrupt cell walls of chickpeas, then starch availability was evaluated by in vitro digestion [22]. The extent of disruption was determined by separating milled material into different particle size fractions. The most disrupted chickpea material (mean particle size < 0.21 mm) had the greatest digestibility (82.9% starch digested) compared to the least disrupted material (mean particle size of 1.85 mm) which had significantly less starch digestibility (33%) [22].

Previous research with tree nuts has also found that cell wall integrity limits availability of nutrients and energy. Processing and mastication, which disrupts cell wall integrity, have been shown to increase availability of fat and energy [4,23,24]. Previously we demonstrated that almond processing affects bioaccessible energy [4]. When comparing different almond forms, metabolizable energy values were significantly lower for processed nuts [4]. Whole natural almonds provided the least metabolizable energy at 4.42 kcal/g, almond butter provided the most metabolizable energy at 6.53 kcal/g, and chopped roasted almonds provided an intermediate value at 5.04 kcal/g [4]. Cassady et al. [23] instructed subjects to chew almonds either 10 times, 25 times, or 40 times before swallowing. Fecal collections demonstrated that fecal fat excretion was inversely associated with mastication, thus absorption was improved by increased mastication. Similarly, Grundy et al. demonstrated that increased mastication reduces almond particle size and increase lipid bioaccessibility [24]. For this study, we did not instruct the participants to masticate in any particular manner so that we could assess their normal consumption style, to more accurately reflect free-living behavior.

The chickpeas and lentils used in this study represented a combination of cultivars reflecting consumption data; thus, differences among cultivars cannot be discerned. Likewise, compositional data found in nutritional databases, including energy, can also represent multiple cultivars, as cultivar information is rarely provided. Future research can focus on measuring the energy value of specific cultivars. The strengths of this study are the adequate sample size based on statistical inference, a representative study cohort of healthy subjects, appropriate adaptation to the diet, and collections of urine and feces that were of sufficient length. Weaknesses include that the study was conducted in a limited number of adults (but with a sample size in accord with our power calculation described in the Methods section) and that energy lost via fermentation was not accounted for, though fermentation is not considered an appreciable route of energy loss. Accounting for gut fermentation would actually lower the metabolizable energy even further. An additional weakness with all free-living human intervention trials is that it is difficult to ensure complete compliance at all times.

## 5. Conclusions

In conclusion, the metabolizable energy in lentils and chickpeas is significantly lower than the values predicted by the Atwater calculations. These results have provided a measurement of metabolizable energy of these pulses as part of a mixed diet consumed by healthy adults. Data on the energy value of these pulse crops is important for accurate food labeling and dietary recommendations geared toward weight management.

## Figures and Tables

**Table 1:** Distribution of lentil and chickpea varieties (% of total weight) in each serving provided to study participants.

Lentil Variety	% of Total	Chickpea Variety	% of Total
Red Lentil		Large Chickpea	
Red Chief	3.43	Sierra	6.91
Cedar	3.43	Nash	6.91
Morton	3.43	Royal	6.91
Small Lentil		Troy	6.91
Pardina	20.10	HB-14	6.91
Medium Lentil		Small Chickpea	
Brewer	22.43	Billy Beans	11.52
Merrit	22.43	Bronic	11.52
Richlea	22.43	Sawyer	11.52
Large Lentil		Canned Chickpea	30.90
Laird	2.30		

**Table 2:** Daily intake of nutrients and energy from controlled diets (*n* = 18).

	Sex ^1^	Base Diet	Base Diet + Chickpea Diet	Base Diet + Lentil Diet	SEM ^2^	*P* ^3^
Dry matter (g)	F	444 ^a^	450 ^b^	466 ^c^	25	<0.0001
	M	578 ^a^	585 ^b^	605 ^c^	37	
Gross energy (MJ)	F	9.69 ^a^	9.67 ^a^	9.92 ^b^	0.54	<0.0001
	M	12.61 ^a^	12.58 ^a^	12.89 ^b^	0.79	
Gross energy (kcal)	F	2316 ^a^	2311 ^a^	2371 ^b^	130	<0.0001
	M	3013 ^a^	3006 ^a^	3081 ^b^	188	
Protein (g) ^4^	F	86.6 ^a^	91.6 ^b^	97.9 ^c^	5.1	<0.0001
	M	112.7 ^a^	119.1 ^b^	127.0 ^c^	7.4	
Fat (g)	F	71.2 ^a^	65.2 ^b^	64.1 ^c^	3.8	<0.0001
	M	82.4 ^a^	85.0 ^b^	83.5 ^c^	5.4	
Total carbohydrate (g) ^5^	F	271 ^a^	278 ^b^	289 ^c^	16	<0.0001
	M	353 ^a^	362 ^b^	375 ^c^	23	
Total dietary fiber (g)	F	25.0 ^a^	32.0 ^b^	49.7 ^c^	2.1	<0.0001
	M	35.9 ^a^	45.9 ^b^	60.5 ^c^	2.9	

^1^ Data from males and females are presented separately to accommodate significant interactions between sex and treatment. ^2^ Standard error of the mean. ^3^ Probability of treatment effect from analysis of variance. If there is a significant (*p* < 0.05) treatment effect, paired comparisons of treatments are indicated with superscripts (rows with different superscripts are different (*p* < 0.05)). See text for additional details on the statistical model and analysis. ^4^ Calculated as nitrogen x 6.25. ^5^ Total carbohydrates determined by difference (see text for details).

**Table 3:** Daily urine and fecal excretion of nutrients and energy and bowel movements (*n* = 18).

	Sex ^1^	Base Diet	Base Diet + Chickpea Diet	Base Diet + Lentil Diet	SEM ^2^	*P* ^3^
Dry matter (g)	F	24.0 ^b^	31.6 ^a^	35.9 ^a^	3.0	0.002
	M	35.3 ^b^	45.3 ^a^	48.5 ^a^	4.3	
Gross energy (kJ)	F	523 ^b^	690 ^a^	757 ^a^	59	0.004
	M	757 ^b^	950 ^a^	1004 ^a^	88	
Gross energy (kcal)	F	125 ^b^	165 ^a^	181 ^a^	14	0.004
	M	181 ^b^	227 ^a^	240 ^a^	21	
Nitrogen (g)	F	1.47 ^a^	2.03 ^b^	2.25 ^b^	0.14	<0.001
	M	2.14 ^a^	2.57 ^b^	3.02 ^c^	0.20	
Fat (g)	F	3.0	3.7	3.0	0.4	0.47
	M	4.1	3.9	3.2	0.6	
Total carbohydrate (g) ^4^	F	8.3 ^b^	11.1 ^b^	14.5 ^a^	1.9	0.009
	M	12.8 ^b^	19.9 ^a^	20.8 ^a^	2.7	
Total dietary fiber (g)	F	7.2	10.7	12.9	1.5	0.24
	M	11.8	15.3	17.5	2.0	
Bowel movements (#/day)	F	1.0	1.2	1.3	0.1	0.18
	M	1.2	1.4	1.2	0.2	

^1^ Data from males and females are presented separately to accommodate significant interactions between sex and treatment. ^2^ Standard error of the mean. ^3^ Probability of treatment effect from analysis of variance. If there is a significant (*p* < 0.05) treatment effect, paired comparisons of treatments are indicated with superscripts (rows with different superscripts are different (*p* < 0.05)). See text for additional details on the statistical model and analysis. ^4^ Total carbohydrates determined by difference (see text for details).

**Table 4:** Apparent digestibility of overall diet components with or without pulses (*n* = 18).

	Base Diet	Base Diet + Chickpea Diet	Base Diet + Lentil Diet	SEM ^1^	*P* ^2^
Dry matter (%)	94.2 ^b^	92.7 ^a^	92.1 ^a^	0.4	0.007
Gross energy (%)	94.3 ^b^	92.7 ^a^	92.3 ^a^	0.4	0.01
Fat (%)	95.7	94.9	95.8	0.4	0.87
Total carbohydrate (%) ^3^	96.7 ^b^	95.3 ^a^	94.7 ^a^	0.4	0.008
Total dietary fiber (%)	68.9	69.6	72.8	2.9	0.45
Nitrogen balance (g/day)	1.25	1.77	2.11	0.41	0.35

^1^ Standard error of the mean. ^2^ Probability of treatment effect from analysis of variance. If there is a significant (*p* < 0.05) treatment effect, paired comparisons of treatments are indicated with superscripts (rows with different superscripts are different (*p* < 0.05)). See text for additional details on the statistical model and analysis. ^3^ Total carbohydrates determined by difference (see text for details).

**Table 5:** Comparison of measured metabolizable energy of chickpeas and lentils to Atwater calculated values.

	Measured Energy Content	Calculated Energy Content by Atwater General Factors ^1^			Calculated Energy Content by Atwater Specific Factors ^2^		
	kJ per Serving ^3,4^	kJ per Serving ^3^	% Difference from Measured	*P* ^5^	kJ per Serving ^3^	% Difference from Measured	*P* ^5^
Chickpeas ^6^	515 ± 17	577	10.4	0.002	561	8.0	0.02
Lentil ^6^	498 ± 17	594	16.0	<0.0001	577	13.6	0.0003

^1^ Using Atwater General Factors of 16.7, 37.7, and 16.7 kJ/g of protein, fat, and carbohydrate, respectively. Calculated using macronutrient composition of pulses fed in this study. ^2^ Using Atwater Specific Factors of 14.5, 35.0, and 17.0 kJ/g of protein, fat, and carbohydrate, respectively. Calculated using macronutrient composition of pulses fed in this study. ^3^ Serving size is 0.5 cup (85.5 g for chickpeas and 98.5 g for lentils). ^4^ Mean ± SEM. ^5^ *p* value of Student’s paired *t*-test. ^6^ *n* = 18.

## Data Availability

The original data presented in the study are openly available in Ag Data Commons at https://agdatacommons.nal.usda.gov/.
